# Are radiologists ready to evaluate true response to immunotherapy?

**DOI:** 10.1186/s13244-021-00968-w

**Published:** 2021-02-24

**Authors:** Inci Kizildag Yirgin, Sukru Mehmet Erturk, Izzet Dogan, Sezai Vatansever

**Affiliations:** 1 Department of Radiology, Oncology Institute, Istanbul University, Istanbul, 34390 Capa Turkey; 2 Department of Radiology, Istanbul Medical Faculty, Istanbul University, Istanbul, 34390 Capa Turkey; 3 Department of Medical Oncology, Oncology Institute, Istanbul University, Istanbul, 34390 Capa Turkey

**Keywords:** Immunotherapy, Tumor response, irRC, irRECIST, iRECIST

## Abstract

**Background:**

Standardized response criteria for evaluating patients radiological imaging have an essential role in oncological management. Immunotherapy, using immune checkpoint inhibitors (ICIs), including drugs targeting cytotoxic T-lymphocyte-associated antigen 4 and programmed cell death protein 1 or its ligand, promise a new role that has demonstrated improvement management in cancers resistant to chemotherapy. This article reviews the literature to understand the most useful response evaluation criteria for optimal patient management under immunotherapy treatment. Areas that warrant further research are described.

**Conclusion:**

In conclusion, ICIs have become more widely accepted and used by medical oncologists. Radiologists face challenges in assessing tumor response and becoming more involved in the management of treatment. The latest published immune-RECIST criteria can be used in response assessment, but further prospective evaluation is needed with registration clinical trials to be definitively validated.

## Key points

- Many criteria have been described since 1979: WHO, RECIST 1.0, RECIST 1.1, irRC, irRECIST, and iRECIST.
- Clinicians and radiologists faced confounding imaging features that they had to identify when ICIs had their place in clinical use. Imaging has a crucial role in treatment planning, local staging, systemic staging by evaluating nodal or distant metastases, and response evaluation to immunotherapy by follow-up images.
- No randomized controlled prospective trials have compared different response criteria for patients under immunotherapy treatment.
- This article reviews the literature to understand the most useful response evaluation criteria for optimal management for patients under immunotherapy treatment.
- The latest published iRECIST criteria can be used in response assessment, but further prospective evaluation is needed with registration clinical trials to be definitively validated.

## Background

Cancer immunotherapy using immune checkpoint inhibitors (ICIs), including drugs targeting cytotoxic T-lymphocyte-associated antigen 4 (CTLA-4) and programmed cell death protein 1 (PD-1) or its ligand, promise a new role that has demonstrated improvement management in cancers resistant to chemotherapy [1–6]. ICIs are used to treat many different types of cancer, including head and neck squamous cell carcinoma, renal cell carcinoma, melanoma, Hodgkin lymphoma, non–small cell lung cancer (NSCLC), and urothelial cancer [7].

Food and Drug Administration (FDA) accepted the ipilimumab treatment for metastatic melanoma in 2011. After that, a significant increase using of ICIs was observed. Clinicians and radiologists faced confounding imaging features to address when these drugs had their place in clinical use. Imaging has a crucial role in treatment planning, local staging, systemic staging by evaluating nodal or distant metastases, and response evaluation to immunotherapy by follow-up images [8].

**Fig. 1:**
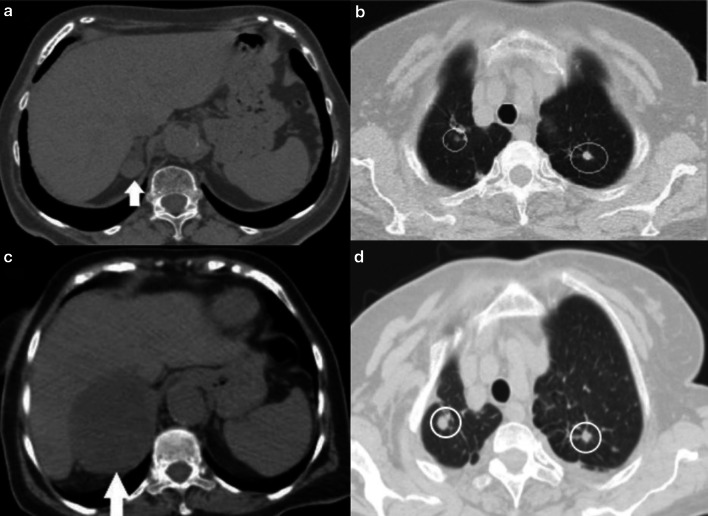
Hyperprogression, a 74-year old female patient with metastatic non-small cell lung cancer under treatment with atezolizumab (anti-PD-L1 monoclonal antibody). **a, b** Before the initiation of immunotherapy, axial CT images show the metastases in the right adrenal gland (arrow) and upper lobes of both lungs (circles). **c** Axial CT image shows an increase in tumor burden of target lesion more than 50% compared with scans done before immunotherapy initiation. **d** There is also an increase in size in non-target lesions in both upper lung lobes(circles)

New response types such as pseudoprogression, hyperprogression, or a dissociative response may not be accurately interpreted with the conventional response criteria [9]. The disease is classified as pseudoprogression when the target lesion continues to grow or the appearance of new lesions followed by shrinkage of tumoral lesions. Biologic hypotheses of enlargement are explained by stimulating the immune system by hyper-activated T cells [10]. In this case, treatment may be terminated early, mistakenly considering that treatment is not effective. The appearance of new lesions or, at least, a 50% increase in total tumor diameters of target lesions is defined as hyperprogression (Fig. 1). Reference imaging must be a pre-treatment imaging done within eight weeks of the immunotherapy initiation [11]. The dissociative response is another response pattern that can be considered progression when using traditional criteria, which means enlargement in the size of some lesions and a reduction in other disease sites simultaneously [12].

**Fig. 2:**
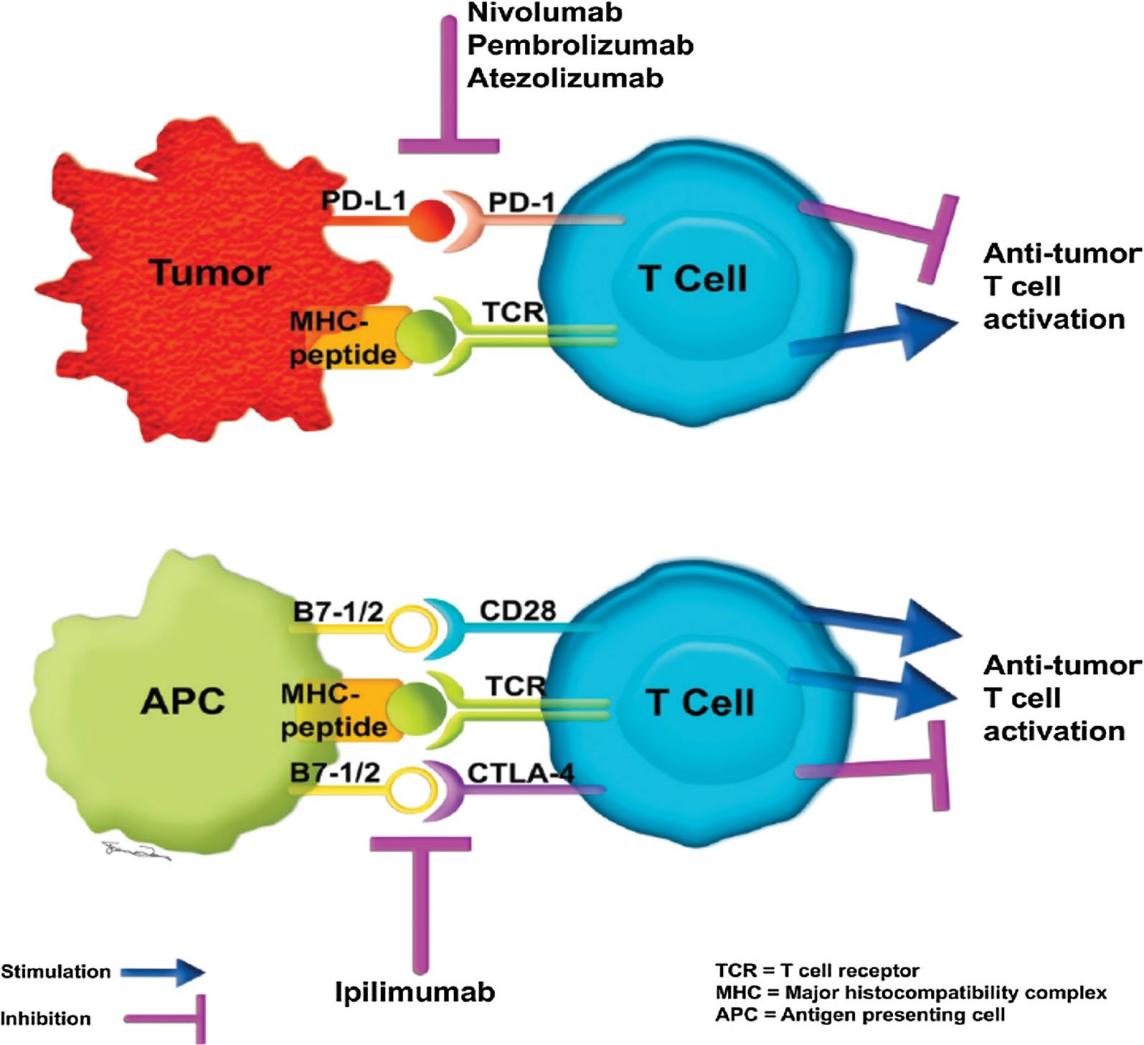
Ilustration shows mechanisms of action of immune check point inhibitors, including anti-CTLA-4 (ipilumumab), anti-PD-1 (nivolumab, pembrolizumab) and, anti-PD-L1 (atezolizumab) monoclonal antibodies (permission obtained from RSNA, Wang GX, Kurra V, Gainor JF, et al. (2017) Immune Checkpoint Inhibitor Cancer Therapy: Spectrum of Imaging Findings. Radiographics 37:2132–44)

The mechanism of immunotherapy has been defined clearly (Fig. 2). Chemotherapeutic agents target critical processes for cell division in rapidly growing and dividing cancer cells. They target cells at different cell cycle phases and cause a cytotoxic effect [13]. In contrast, immunotherapy stimulates the immune system, helps increase the amount of cytotoxic T- lymphocytes. To understand how immunotherapy works, we need to start with antigens and proteins on cytotoxic T-lymphocytes. These cells mainly contain CTLA-4 antigen and programmed cell death ligand-1 (PDL-1) receptors on their cell membranes, known as immune checkpoints [14]. Both inhibit T-cells activity through different mechanisms to suppress autoimmunity and check the immune system responses. CTLA-4 inhibits T-cells' activation by Antigen-presenting cells (APCs) antigens, decreasing clonal proliferation of tumor-specific T- cells. The activations of effector T- cells reduce with the binding of PD-L1 on the tumor cell membrane and PD-1 on the T- cell [15]. ICIs assist the immune system in accepting cancer cells as unknown for the body by blocking these bindings. Three main types of drugs– CTLA-4 antibodies (Ipilimumab), PD-1 antibodies, and PD-L1 antibodies (Nivolumab, Pembrolizumab, Atezolizumab) have been approved by the FDA [16].

No randomized controlled prospective trials have compared different response criteria for patients under immunotherapy treatment. It is necessary to systematically characterize tumor response during therapy, identify different patterns, and investigate their association with survival. This article reviews the results of studies in the literature to clarify suggestions for managing patients imaging features.

## Clinical question

**Fig. 3:**
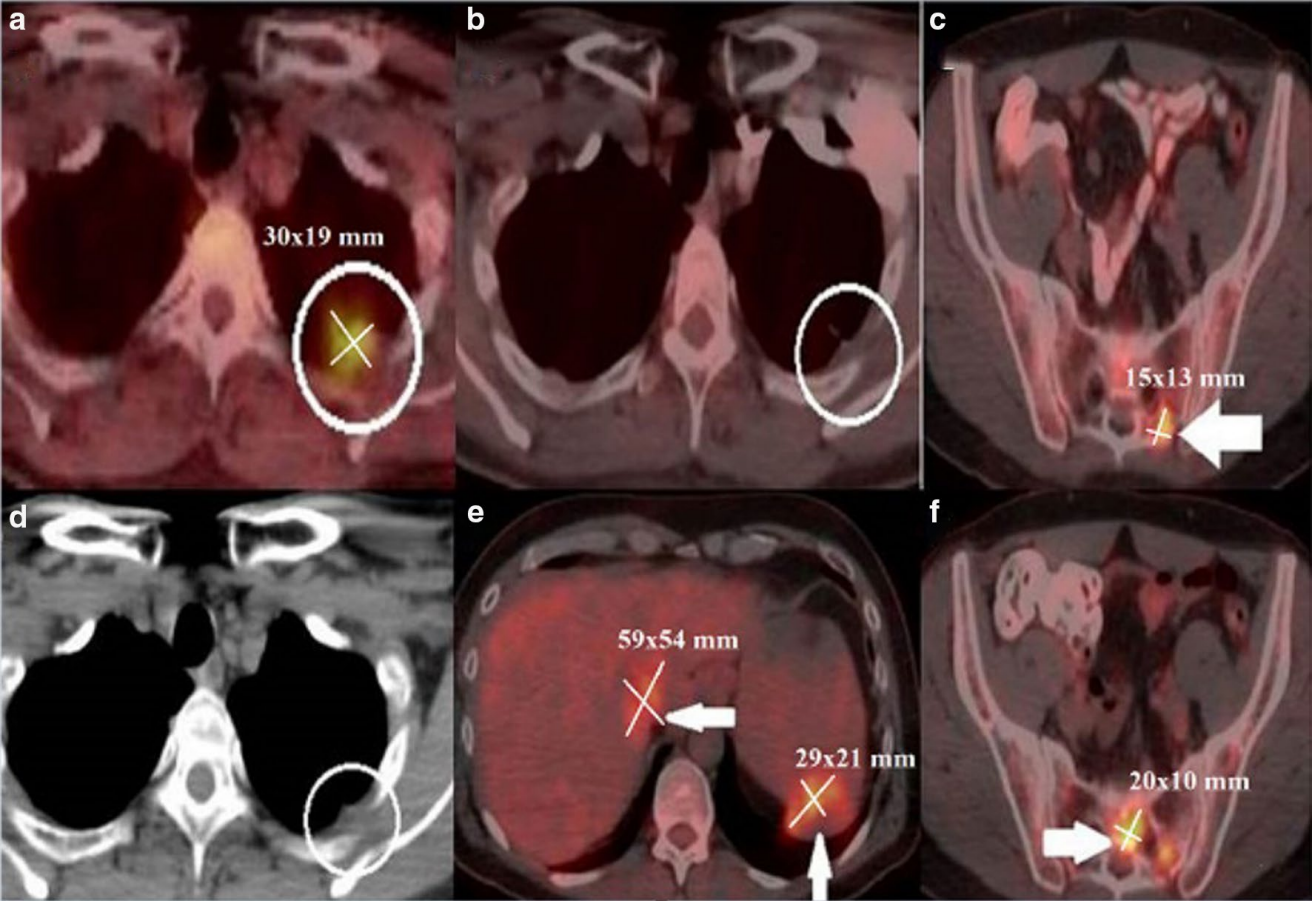
**a** A 44-year-old man diagnosed with metastatic malignant melanoma presented with a new metastasis measured 30 × 19 mm in diameter (circle) in the upper lobe of the left lung while under the temozolomide and cisplatin treatment. **b, c** In the first control performed after 12 weeks initiation of immunotherapy with nivolumab (anti-PD-1 monoclonal antibody), the lesion in the left lung was almost regressed (circle), but there was a new bone metastasis measured 15 × 13 mm in diameter in the sacrum (arrow). **d-f** In the second control performed after 16 weeks, the lesion in the left lung was regressed entirely (circle). Many new metastases were detected in the liver, spleen, and sacrum, measured 59 × 54 mm, 29 × 21 mm, and 20 × 10 mm in diameters, respectively (arrows)

A new metastasis in the upper lobe of the left lung of a 44-year-old man with metastatic malignant melanoma was detected while he was under temozolomide and cisplatin treatment. Immunotherapy treatment was initiated due to his progressive disease, and nivolumab treatment started until a new progression. In the first control performed after 12 weeks, the left lung lesion was regressed, but there was a new bone metastasis in the sacrum. The response was accepted as a partial response according to the immune-related response criteria (irRC) and immune-related RECIST (irRECIST) while classified as unconfirmed progressive disease (UPD) according to immune-RECIST (iRECIST). Immunotherapy treatment continued, and the second control was performed 16 weeks later. In the second control, many new metastases were detected in the liver, spleen, and sacrum, and the response was evaluated as progressive disease (PD), according to the irRC and irRECIST and as confirmed progressive disease (CPD) according to the iRECIST (Fig. 3 and Table 1).

**Table 1:** Measurements of Fig. 3

Target lesions	irRC	irRECIST	iRECIST
Baseline: 30 × 19 mm	30 × 19 = 570	30	30
First control: 15 × 13 mm	15 × 13 = 195 PR (%65)	15 PR (%50)	15 UPD (new lesion)
Second control: 15 × 13 mm, 59 × 54 mm, 29 × 21 mm and 20 × 10 mm	(15 × 13) + (59 × 54) + (29 × 21) + (20 × 10) PD (> %25)	15 + 59 + 29 + 20 PD (> %20)	CPD (3 new lesion)

### The imaging question and criteria for evaluating tumor response and different measurement details with illustrative images

Standardized response criteria for evaluating patients' radiological imaging have an essential role in oncological management. Many criteria have been described since 1979 include WHO (World Health Organization), RECIST 1.0 (Response Valuation Criteria in Solid Tumors), RECIST 1.1, irRC, irRECIST, iRECIST. The criteria for evaluating control images vary depending on whether the patient's treatment is chemotherapy or immunotherapy. Although the use of RECIST 1.1 criteria has been accepted widely in evaluating patients receiving chemotherapy, there is no consensus in the literature for assessing patients receiving immunotherapy. Many questions are raised regarding how we currently manage the response. Which response criteria correlate better with patient prognosis? How to assess newly detected lesions? Should we compare the total tumor burden with the previous control or before the start of treatment? This article reviews the literature to understand the most useful response evaluation criteria for optimal management for patients under immunotherapy treatment.

There are multiple dissimilarities between conventional response criteria and immune response criteria (Table 2). The first criteria described by WHO in 1979 introduced a bidimensional measurement method of tumor lesions and calculated the sum of the products (SPD) as tumor burden [17]. According to the WHO criteria, treatment efficacy was categorized build on measurement changes compared to images obtained before therapy initiation. The four major responses were determined as follows, complete response (CR), defined as the extinction of all malignant lesions; partial response (PR), defined as ≥ 50% reduction in the SPD; PD, defined as a ≥ 25% enlargement in the dimension of 1 or more lesions, or the emergence of novel tumoral lesions; and stable disease (SD), defined when no more than ≥ 50% decrease in SPD observed or more than ≥ 25% increase in at least one lesion size. However, it was observed that the WHO criteria could not answer some of the critical questions identified as they started to be used. The measurable and nonmeasurable lesions were not classified, and the minimum dimension and the maximum number of lesions taken into account were not clear [18, 19]. U.S. National Cancer Institute, European Organization for Research and Treatment of Cancer, and WHO revised the WHO criteria and published new guidelines, namely the RECIST, in 2000 [20]. With the introduction of RECIST criteria, the unanswered questions became addressed. In the response assessment, the minimum dimension of measurable lesions was 10 mm, and the highest number of target lesions was 10. Sum of the longest diameters (unidimensional) of target lesions replaced SPD. CR was defined as extinction of all lesions, PR was defined as a ≥ 30% decrease in the sum of the longest diameters (SLD) of target lesions, and PD as a ≥ 20% increase in the SLD or detection of one or more new lesions or evident growth of non-target lesions. SD was reserved for patients without CR, PR, or PD. A new version of RECIST, RECIST 1.1, was published in 2009. It added the revised evaluation of new lesions, cystic and necrotic lesions and lymph nodes, bone lesions [21, 22]. Categories based on percentage changes were the same as the previous version. Tumor response was based on the measurement of five lesions (instead of ten). Measurements included the maximum diameter in the longitudinal axis for non-nodal lesions and the maximum diameter in the short axis for nodal lesions. Lymph nodes with a short-axis diameter of at least 10 mm but smaller than 15 mm are considered non-target lesions, and lymph nodes with a short-axis diameter of 15 mm or larger are considered target lesions [23].

**Table 2:** Comparison of RECIST 1.0, RECIST 1.1, irRC, irRECIST, and iRECIST

	CR	PR	SD	PD	Confirmation of PD	New lesions
RECIST 1.0 Unidimensional > 10 mm 10 lesions in total, 5 per organ	Disappearance of all lesions	> 30% decrease from baseline	Neither CR nor PD	20% increase; no CR, PR, or SD documented before increased disease	Not applicable	PD
RECIST 1.1 Unidimensional > 10 mm 5 lesions in total, 2 per organ	Disappearance of all lesions	> 30% decrease from baseline	Neither CR nor PD	> 20% increase in the nadir of the sum of target lesions (with a minimum a of 5 mm)	Not applicable	PD
irRC Bi-dimensional 5 mm × 5 mm 15 lesions in total, 5 per organ	Disappearance of all lesions	> 50% decrease from baseline	Neither CR nor PD	> 25% increase in the nadir of the sum of target lesions	At least 4 weeks later	Incorporated in the sum of measurements
irRECIST Unidimensional > 10 mm 5 lesions in total, 2 per organ	Disappearance of all lesions	> 30% decrease from baseline	Neither CR nor PD	> 20% increase in the nadir of the sum of target lesions (with a minimum a of 5 mm)	At least 4 weeks after and up to 12 weeks	Incorporated in the sum of measurements
iRECIST Unidimensional > 10 mm 5 lesions in total, 2 per organ	Disappearance of all lesions	> 30% decrease from baseline	Neither CR nor PD	> 20% increase in the nadir of the sum of target lesions (with a minimum a of 5 mm)	At least 4 weeks after and up to 8 weeks	iUPD; not incorporated in the sum becomes iCPD if confirmed

RECIST, response evaluation criteria in solid tumors; irRC, immune-related response criteria; irRECIST, immune-related RECIST; iRECIST, immune RECIST; CR, complete response; PR, partial response; SD, stable disease; PD, progressive disease; iUPD, immune unconfirmed progressive disease; iCPD, immune confirmed progressive disease

**Fig. 4:**
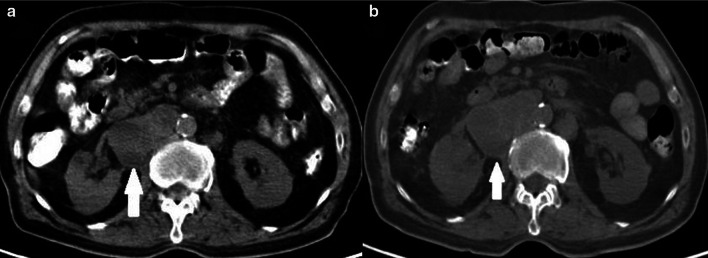
Stable disease, a 70-year old male patient with metastatic renal cell cancer under treatment with nivolumab (anti-PD-1 monoclonal antibody). **a** Axial CT image shows right paraaortic conglomerated mass like pathological lymph nodes measured 61.4 mm in diameter. **b** In the control CT examination performed even after one year, the size of the conglomerated mass like pathological lymph nodes were stable and measured 65.4 mm in diameter

**Fig. 5:**
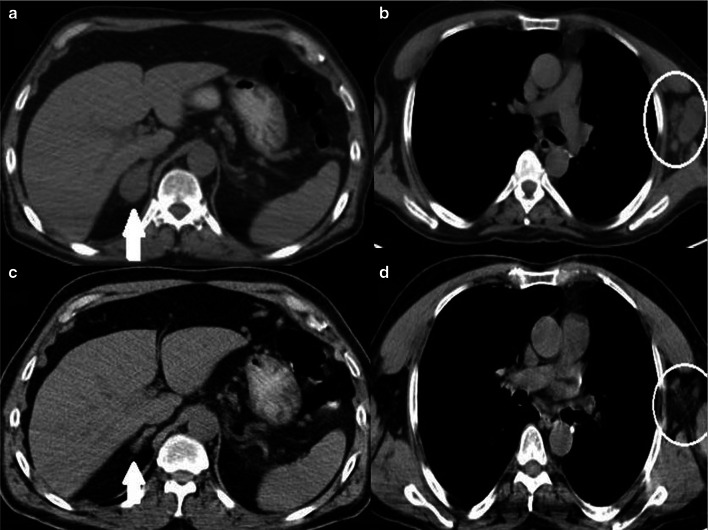
Complete response, a 74-year old male patient with metastatic non-small cell lung cancer under treatment with nivolumab (anti-PD-1 monoclonal antibody). **a, b** Axial CT images show metastasis in the right adrenal gland (arrow) and multiple metastatic lymph nodes in the left axilla (circle). **c, d** There are no lesions higher than > 10 mm in both regions in the control images performed eight weeks after treatment initiation

Up to now, our text was about the criteria used when evaluating cytotoxic chemotherapy treatment. However, the variety of apparitions of successful treatment response after immune therapy is different. Five significant distinctions in tumor burden response to immunotherapeutic agents compared to cytotoxic agents are discussed by expert panelists in 2004 and 2005 [24, 25]. These conversations resulted in these five outcomes (I) time needed to pass to occur the anti-tumoral effectiveness may be longer for immunotherapeutic agents compared to cytotoxic agents; (II) good responses to immunotherapeutic agents can be verified even after PD; (III) to interrupt using immunotherapeutic agents cannot be relevant for some patients, unless PD is confirmed; (IV) toleration for "clinically negligible" PD (for instance, appearing small new lesions with of reduction in other lesions) is advised; and (V) continued stable disease (SD) might mean the being of anti-tumoral effect. The expert panel the results of the Phase II clinical trial of 227 patients with advanced melanoma treated with CTLA-4 inhibitor (Ipilimumab) agent and defined a new response assessment criteria, the irRC that they developed from WHO criteria [25]. Like WHO criteria, the four major response evaluations were determined as follows, CR, defined as the extinction of all malignant lesions; PR, defined as ≥ 50% reduction in the SPD; PD, defined as a ≥ 25% increase in SPD; and SD, when no more than ≥ 50% decrease in SPD can be observed or more than ≥ 25% increase in at least one lesion size. SD is a response pattern that does not demonstrate major differences when applied to all immune-response criteria (Fig. 4). CR, PR, and PD need to be confirmed at four weeks. As a result of the studies, it was determined that the new lesions were not evaluated in favor of progression, as in the RECIST criteria, and the definition of "clinically insignificant" new lesions developed with irRC [25, 26]. New lesions are added to the total tumor burden, and the number of lesions to assess was increased compared to RECIST 1.1 (up to 5 per organ, up to 10 visceral in WHO *vs.* 2 per organ, 5 in total in RECIST 1.1). Bidimensional measurements on the long and short axes were performed. Despite its rational approach regarding the new lesions, irRC was widely criticized. First, two-dimensional evaluation is more challenging to apply than one-dimensional evaluation; a higher number of target lesions causes more time to be spent in daily work; third, lymph nodes evaluation is not determinedly explained [24, 27]. Nishino and Coll developed new immune-related criteria named irRECIST in 2013, combining RECIST 1.1 with the newly determined rules of irRC; they aimed to create a faster and more user-friendly reporting system [28]. The minimum longest diameter must be at least 10 mm for visceral target lesions and 15 mm for target lymph node in the short axis. Unidimensional measurements were performed. Similar to RECIST 1.1 criteria, the four major response groups were defined as CR (evanescence of all malignant lesions with lymph nodes reduced to less than 10 mm in short-axis), PR, (≥ 30% reduction in the sum of diameters (SOD) compared to baseline, without new lesions), PD (a ≥ 20% growth in SOD or ≥ 5 mm absolute growth in SOD), and SD (when PR or PD can not be confirmed). Images of a patient with metastatic non-small cell lung cancer under treatment with nivolumab show no lesions higher than > 10 mm in all regions in the control images performed eight weeks after the initiation of treatment and assessed as CR with all immune response criteria (Fig. 5). The number of target lesions to evaluate has been determined as 5 in total, with no more than two lesions per organ. Significant differences between RECIST 1.1 and irRECIST are in evaluating new lesions. Measurable new lesions are added in SOD in the irRECIST while accepted as PD with RECIST criteria. Unmeasurable new lesions like bone and leptomeningeal metastases; malignant free fluid in the abdominal cavity inflammatory breast cancer; pleural and pericardial effusions; lesions with cystic nature; dermal lesions and lymphangitic carcinomatosis were recorded separately. Verification of CR, PR, and PD by a sectional radiological method minimum four weeks later is another difference from RECIST 1.1.

**Fig. 6.**
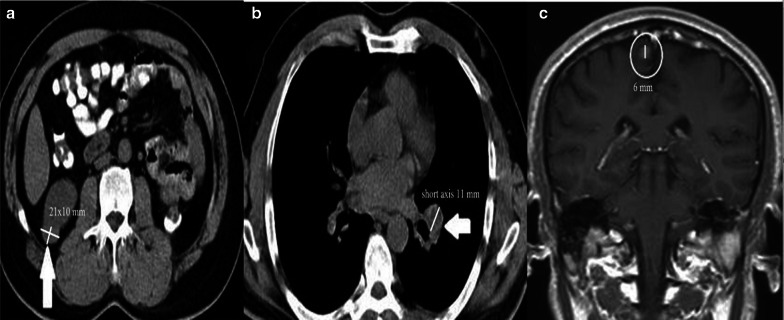
52- year old male patient diagnosed with metastatic renal cell carcinoma. **a, b** Axial CT images show hypodense malignant lesion (arrow) in the lower pole of the right kidney and pathological lymph node in the left hilar region (arrow). **c** Coronal post-contrast T1 weighted MR image shows metastasis in the right parietal lobe (circle). Chemotherapy was started

**Fig. 7:**
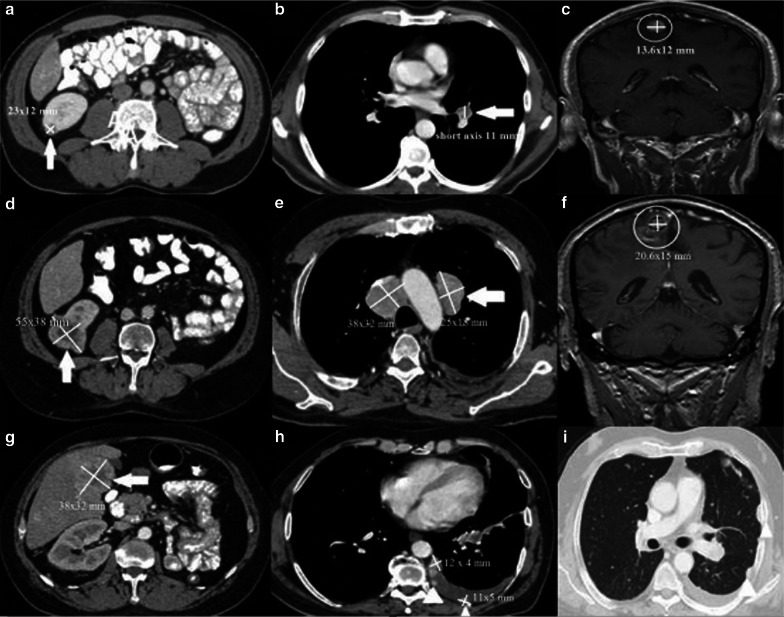
Continue from Fig. 6. **a–c** Neither CR nor PD was observed in the first control after initiation of chemotherapy treatment (arrows and circle). Immunotherapy treatment was started after this control imaging findings. **d–f** > 20% increase in the nadir of the sum of target lesions was observed (arrows and circle) **g-i** At the same control, new lesions were developed. Axial CT image shows a metastatic lesion in the right lobe of the liver (arrow) and multiple metastatic nodules in the left parietal pleura (arrowheads)

A new response criterion called iRECIST developed by the RECIST working group settled on principles of RECIST 1.1 to provide consistent data collection in clinical trials based on immunotherapeutic in 2017 [29]. There are no differences between this new criteria and the RECIST 1.1 and irRECIST on suggested radiological modalities for imaging evaluation, describing measurable and unmeasurable lesions. Categories based on percentage changes are different. The five major responses were determined as follows, immune complete response (iCR), immune partial response (iPR), immune stable disease (iSD), unconfirmed progressive disease (iUPD), and immune confirmed progressive disease (iCPD). The disappearance of all malignant lesions with lymph nodal shorter-axis reduced to less than 10 mm and apparent no new lesions called iCR, ≥ 30% reduction in the SOD relative to baseline with no new lesions and undetermined increase of non-target lesions called iPR, increase ≥ 20% of the SOD relative to baseline or ≥ 5 mm absolute increase in SOD or increase of non-target lesions or appearance of new lesion called iUPD, verification is required at least 4–8 weeks later than the first evaluation. Development of another new lesion, increased size of the target or non-target lesions; progression in the sum of new target lesions > 5 mm; increase of the non-target lesions; defined as iCPD, and no definition for iCR/iPR/iUPD/ iCPD categorized iSD. Evaluation of a patient (Figs. 6, 7) diagnosed with metastatic renal cell carcinoma and treated by chemotherapy and immunotherapy with different response criteria are detailed in Table 3.

**Table 3:** Measurements of Figs. 6 and 7

Target lesions	RECIST	irRC	irRECIST	iRECIST
Baseline: right kidney: 21 × 10 mm, lymph node: nontarget (< 15 mm in short axis), right parietal lobe: non -target (< 10 mm in long axis)				
First control: Right kidney: 23 × 12 mm, lymph node: non-target, right parietal lobe: 13.6 × 12 mm	SD	Not for chemotherapy treatment response evaluation	Not for chemotherapy treatment response evaluation	Not for chemotherapy treatment response evaluation
Second control: Right kidney: 55 × 38 mm, right parietal lobe 20.6 × 15 mm	PD	PD (> %25 increase in total tumor burden)	PD (> %20 increase in total tumor burden)	iUPD Need to be confirmed at 4–8 weeks
Target new lesions: Right liver lobe:38 × 32 mm, lymph nodes:25 × 18 mm and 28 × 39 mm pleural nodules:12 × 4 mm and 11 × 5 mm				
Non-target new lesions: Pleural effusion and < 10 mm pleural nodules				

### Studies contributing to this historical development

In 2009 Wolchok et al. published a guideline [25] for irRC based on the multinational study that included 487 patients diagnosed with advanced melanoma and treated by anti-CTLA-4 (ipilimumab) [30–34]. Tumor assessments were evaluated at the 12th week, the end of the first cycle of treatment. Treatment was continued in cases with PD according to WHO criteria without rapid clinical worsening before the 12th week to take into account, enough duration for immune activation following antitumor responses while was discontinued due to drug intolerance or withdrawal of consent. Previous data showed approximately 60% of responses (PR/CR) were detected at the 12th week of treatment initiation [35–37]. In 2005, Maker et al. reported that some cases treated with ipilimumab showed progression in total tumor burden or development of new lesions before a treatment response with a phase I/II study [38]. At the same time, Wolchok et al. formed two groups (first group of patients categorized in CR, PR, or SD and second group of patients categorized in PD) according to WHO criteria while evaluating the studies. Their data recommend that patients in both groups have equivalent survival. When the same groups were reevaluated according to irRC criteria, at least 10% of patients with PD (according to the WHO) had better survival [25]. These results showed that the addition of new lesions to the total target lesion diameter, which was considered progression immediately according to WHO criteria, was more consistent with the patient's clinical data. Scientists realized that the immunotherapy response criteria should be different, should have less misclassification. Further studies need to be conducted on this topic.

Nishino et al. published a phase II multicenter research that included 57 patients diagnosed with unresectable stage III or stage IV malignant melanoma treated with ipilimumab in 2013 [28]. They hypothesized that using unidimensional measurements could have the same results with bidimensional measurements due to more straightforward and more reproducible measurements. They retrospectively reviewed CT images at baseline and at least one follow-up and calculated findings according to the irRC and irRECIST criteria. Results were highly concordant in the first three follow-up scans, with a Spearman correlation coefficient of 0.959. Only four patients had discordant categories, including 3 with PD by irRC and SD by irRECIST and one with SD by irRC and PD by irRECIST. Most patients (41 of 57, 72%) had SD by both assessments. This study was an essential step in optimizing response evaluation and creating "common language" for further studies, despite including a relatively small number of patients and giving no results about association clinical outcome and response assessment. Since these criteria were not a formal guideline, RECIST 1.1 was continued to be used for evaluation of immunotherapy treatment. In 2016 Jonathan et al. published a phase II global, multicenter trial to confirm the antitumor effect of atezolizumab (anti- PD-L1) in 310 patients diagnosed with advanced urothelial cancer whose disease had increased after prior chemotherapy [39]. They measured the objective response rate by RECIST 1.1 and iRECIST. The objective response rate was 15% (95% CI 11 to 19), with CR observed in 15 patients (5%). Response rates measured by iRECIST were similar to RECIST 1.1. Additionally, 121 patients were continued to treat, although observed progression before the 12th week. In 21 of these patients, target lesion reduction of at least 30% from their baseline scans were observed. This atypical response, called pseudoprogression, suggested that changes should be made in RECIST 1.1. In 2016, Hodi et al. published a phase Ib study called KEYNOTE-001 and evaluated atypical response types and the correlation between overall survival and best overall response measured per irRC and RECIST 1.1 in patients diagnosed with advanced melanoma and used pembrolizumab [40]. Twenty-four (7%) of 327 patients had pseudoprogression in this study. Patients with nonprogressive disease (n = 331) had a 77.6% two-year overall survival rate per both criteria. The survival rate of progressive disease (n = 84) was 37.5% with RECIST1.1, but the same group was categorized in nonprogressive disease with irRC. The survival rate of progressive disease per both criteria (n = 177) was 17.3%. In the survival analysis, pembrolizumab's benefit was ignored in almost 15% of patients by evaluating RECIST 1.1, so early discontinuation of treatment might be possible. The study of Hodi et al. also showed that RECIST 1.1 criteria were insufficient in evaluating immunotherapy treatment. Nevertheless, iRECIST is not yet validated, and it is not recommended for registration trials.

### Evidence-based guideline

The revised RECIST 1.1 guideline and accompanying articles were published in the European Journal of Cancer (EJC) special issue in January 2009 [21]. RECIST 1.1 is the formal and validated guideline for assessment of response for both immunotherapy and chemotherapy treatments. The iRECIST guideline was published in The Lancet Oncology in March 2017. [29]. The iRECIST is a consensus guideline established by the RECIST Working Group, pharma, regulatory authorities, and academia to provide convenient design and documentation for prospectively build a data store to be used to confirm iRECIST or reform RECIST.

### Topics to be investigated

To date, after the introduction of immunotherapy, many questions about response evaluation are still unanswered. Radiological response criteria have been based on studies using drugs with only anti-CTLA4 or anti-PD1/PD-L1 effects. Moreover, enough evidence has not been published whether they are appropriate to evaluate the response of disease treated with different immune checkpoint inhibitors, combinations of them or combinations of immunotherapy and chemotherapy or target therapy. Another missing point in the literature is that it is unclear which criteria can be used in patients receiving both immunotherapy and radiotherapy treatments. Considering that radiotherapy treatment may cause abscopal effects that refer to localized radiation's ability to increase systemic antitumor effects, it can become unclear whether the treatment response depends on immunotherapy or radiotherapy [41].

Although pseudoprogression is one of the most widely written atypical response patterns in the literature, it is rare, occurring in less than 10% of patients [42]. Most patients with increment of total tumor burden or development of new lesions have real PD. Further research is needed to understand immune response mechanisms to predict atypical response patterns (pseudoprogression, hyperprogression, dissociative response). The answer to these points is particularly crucial because the keeping of an inadequate therapy can retard salvage therapy, and keeping therapy in patients with the real progressive disease might leave unprotected them to unwanted side effects of drugs [19].

Until now, we have no clear information about a perfect imaging technique for the assessment of total tumor burden in patients who received immunotherapy. The most common radiological imaging methods are computerized tomography (CT) and magnetic resonance imaging (MRI) used in daily practice. New techniques, including diffusion, perfusion, and metabolic imaging, are needed for a correct diagnosis of accurate progression or inflammatory response. New parts may need to be added to the evaluation criteria after studies on these methods have been carried out.

## Summary

In conclusion, immunotherapy has become more thoroughly accepted and used by medical oncologists. Radiologists face challenges in assessing tumor response and becoming more involved in the management of treatment. The latest published iRECIST criteria can be used in response assessment, but further prospective studies are needed to validate them. Studies focusing on the differences between RECIST 1.1 and iRECIST will improve yhe current practice.

## Data Availability

Not applicable.

## Abbreviations

APCs: Antigen-presenting cells
CPD: Confirmed progressive disease
CT: Computerized tomography
CTLA-4: Cytotoxic t-lymphocyte-associated antigen 4
FDA: Food and drug administration
ICIs: Immune checkpoint inhibitors
iCPD: Immune confirmed progressive disease
Icr: Immune complete response
iPR: Immune partial response
iRECIST: Immune-recist
irRC: Immune-related response criteria
irRECIST: Immune-related recist
iSD: Immune stable disease
iUCPD: Immune unconfirmed progressive disease
MRI: Magnetic resonance ımaging
NSCLC: Non–small cell lung cancer
PD: Progressive disease
PD-1: Programmed cell death protein 1
PDL-1: Programmed cell death ligand-1
RECIST 1.0: Response valuation criteria in solid tumors
SLD: Sum of the longest diameters
SOD: Sum of diameters
SPD: Sum of the products
UPD: Unconfirmed progressive disease
